# Escores Angiográficos na Predição de No-Reflow, a Injuria Miocárdica pode não se Encerrar com a Reperfusão

**DOI:** 10.36660/abc.20210114

**Published:** 2021-03-03

**Affiliations:** 1 Hospitais Aliança, São Rafael Cardiopulmonar – Rede D’Or SalvadorBA Brasil Hospitais Aliança, São Rafael, Cardiopulmonar – Rede D’Or, Salvador, BA - Brasil.; 2 Hospital Ana Nery SalvadorBA Brasil Hospital Ana Nery, Salvador, BA - Brasil.; 3 Universidade Federal da Bahia Hospital Universitário Professor Edgard Santos SalvadorBA Brasil Universidade Federal da Bahia, Hospital Universitário Professor Edgard Santos, Salvador, BA - Brasil.

**Keywords:** Intervenção Coronária, Percutânea/métodos, Infarto do Miocárdio, Aterosclerose, Trombose, Placa Aterosclerótica, Embolização Terapêutica

O infarto com supradesnivelamento de segmento ST (IAMCSST) geralmente é precipitado pela ruptura ou erosão de uma placa aterosclerótica e consequente formação de trombo oclusivo. A intervenção coronária percutânea precoce é o tratamento de escolha por propiciar uma revascularização mais completa e menores complicações do tipo sangramento quando comparado à fibrinólise.^1^^,^^2^

Nas últimas décadas observamos uma evolução substancial no tratamento farmacológico e invasivo do IAMCSST, o que reduziu significativamente a mortalidade precoce. Diversas variáveis influenciam os desfechos clínicos, entre elas, idade do paciente, tempo para reperfusão, complexidade angiográfica e a ocorrência ou não do fenômeno de *no-reflow* (NR) durante o tratamento percutâneo.^3^^,^^4^

NR é definido por inadequada perfusão miocárdica em determinado território, na ausência de obstrução mecânica da coronária epicárdica^5^ e está associado a pior prognóstico clínico.^6^^,^^7^

Nessa edição dos Arquivos Brasileiros de Cardiologia o artigo “O Escore Gensini e a carga trombótica adicionam valor preditivo ao escore SYNTAX na detecção de *no-reflow* após o infarto do miocárdio”, no qual os autores avaliaram a angiografia de 481 pacientes admitidos por IAMCSST e calcularam os escores SYNTAX, Gensini modificado, além da avaliação da carga trombótica de forma objetiva. Foi encontrada uma melhor acurácia da predição do fenômeno NR quando utilizada a combinação das três avaliações.^8^

O escore de Gensini foi descrito pela primeira vez em 1975, leva em consideração três parâmetros para cada lesão coronária: gravidade da obstrução, multiplicada por um fator de acordo com importância da região irrigada pela artéria e ajustada pela presença de colaterais;^9^ contempla estenoses menores que 25% e é, portanto, mais sensível a obstruções parciais que o SYNTAX SCORE. A quantificação objetiva da carga trombótica é determinada pela escala TIMI de 0 a 5, no qual 0 é a ausência de trombo e 5 é a presença de trombo oclusivo.^10^

Relacionar esses escores angiográficos com a presença de fenômeno de NR faz sentido fisiopatológico, pois apesar de não ser totalmente esclarecido, o fenômeno de NR em pacientes submetidos à intervenção percutânea primária tem como uma das causas a microembolização distal,^11^ que por sua vez depende das variáveis angiográficas estudadas pelos autores. No estudo publicado não foram reportados dados sobre os procedimentos (tromboaspiração, *stents*, pós-dilatação), medicações adjuvantes e reversibilidade do fenômeno que também impactam significativamente no prognóstico angiográfico e clínico.

Predizer um fenômeno potencialmente catastrófico ganha importância quando impacta em modificação de conduta antes deste ocorrer. O estudo DEFER-STEMI, publicado em 2014, tocou justamente neste ponto: um *trial* prova de conceito que avaliou o impacto de atrasar o implante de *stent* (com a artéria já reperfundida por balão ou tromboaspiração) com objetivo de reduzir a incidência de NR e o tamanho do infarto, avaliado pela ressonância magnética - o racional é que adiar o implante do *stent* pode dar tempo para ação das drogas antitrombóticas, redução da carga de trombo e consequentemente menos NR e menor área infartada. De fato, nesse estudo, houve uma redução significativa de NR (de 14% para 2% no grupo stent-adiado) e melhora do índice de recuperação miocárdica em seis meses.^12^ Mais tarde, em 2017, uma metanálise reuniu 9 estudos e essa redução de NR não foi observada, contudo, uma melhora da função ventricular de longo prazo no grupo *stent* adiado foi apontada.^13^

Outras estratégias como utilização de drogas intracoronárias (adenosina, bloqueadores do canal de cálcio e nitropussiato) e de inibidores da glicoproteína IIb/IIIa mostraram algum benefício na prevenção e tratamento do NR e necessitam de maiores estudos.^14^

O fenômeno de NR é o maior desafio da reperfusão primária e apesar dos esforços, o conhecimento pouco evoluiu no tratamento ou prevenção dessa condição. Escores angiográficos tornam a avaliação da cineangiocoronariografia mais objetiva e, como demonstrado nesse manuscrito, a associação de escores clássicos (SYNTAX, Gensini, Carga trombótica) colabora na discriminação dos pacientes com pior prognóstico, potencialmente implicando em condutas a serem tomadas para otimizar o tratamento e melhorar desfechos.
