# Supplementary material for: Escores Angiográficos na Predição de No-Reflow, a Injuria Miocárdica pode não se Encerrar com a Reperfusão
Source: Arq Bras Cardiol. 2021 Mar 3;116(3):473–4. [Article in Portuguese] doi: 10.36660/abc.20210114 (PMC8159565; doi:10.36660/abc.20210114)
Supplement: Translation

# Translation: Angiographic Scores in Prediction of No-Reflow, Myocardial Injury May not end with Reperfusion

**Affiliations:** 1 SalvadorBA Brazil Hospitais Aliança, São Rafael, Cardiopulmonar – Rede D’Or, Salvador, BA - Brazil.; 2 SalvadorBA Brazil Hospital Ana Nery, Salvador, BA - Brazil.; 3 SalvadorBA Brazil Universidade Federal da Bahia, Hospital Universitário Professor Edgard Santos, Salvador, BA - Brazil.

**Keywords:** Percutaneous Coronary Intervention/methods, Myocardial Infarction, Atherosclerosis, Plaque Atherosclerotic, Embolization Therapeutic

The ST segment elevation infarction (STEMI) is usually precipitated by the rupture or erosion of an atherosclerotic plaque and the consequent formation of an occlusive thrombus. Early percutaneous coronary intervention is the treatment of choice for providing a more complete revascularization and less bleeding complications when compared to fibrinolysis.^1^^,^^2^

In the last decades, we observed a substantial development in the pharmacological and invasive treatments of STEMI, which significantly reduced early mortality. Several variables affect clinical outcomes, including patient age, time to reperfusion, angiographic complexity and the occurrence or not of the no-reflow (NR) phenomenon during percutaneous intervention.^3^^,^^4^ NR is defined by inadequate myocardial perfusion in a given territory, in the absence of mechanical obstruction of the epicardial coronary^5^ and is associated with a worse clinical prognosis.^6^^,^^7^

In this edition, the “Arquivos Brasileiros de Cardiologia” publish the article “*Gensini Score and Thrombus Burden Add Predictive Value to the SYNTAX Score in Detecting No-Reflow after Myocardial Infarction*”, the authors evaluated the angiography of 481 consecutive patients admitted by STEMI and calculated the SYNTAX and modified Gensini scores, in addition to assessing thrombotic burden objectively. A better accuracy of the prediction of the NR phenomenon was found when the combination of the three scores was used.^8^

The Gensini score was first described in 1975 and takes into account 3 parameters for each coronary lesion: severity of the obstruction, multiplied by a factor according to the importance of the region irrigated by the artery and adjusted by the presence of collaterals;^9^ includes stenoses less than 25% and is, therefore, more sensitive to partial obstructions than SYNTAX SCORE. The objective quantification of thrombotic load is determined by the TIMI scale from 0 to 5, where 0 is the absence of a thrombus and 5 is the presence of an occlusive thrombus.^10^

The relationship of these angiographic scores to the presence of NR phenomenon makes physiopathological sense, because although it is not fully clarified, the NR phenomenon in patients undergoing primary percutaneous intervention has distal microembolization as one of its causes,^11^ which depends on the angiographic variables studied by the authors. In the published study, no data were reported about the procedures (thrombus aspiration, stents, post-dilation), medications used and reversibility of the phenomenon that also significantly impact the angiographic and clinical prognosis.

The act of predicting a potentially catastrophic phenomenon is important when it impacts on changing strategy before it occurs. The DEFER-STEMI study, published in 2014, touched precisely on this point: a proof-of-concept trial that assessed the impact of delayed-stent (with the artery already reperfused by balloon or thrombus aspiration) in order to reduce the incidence of NR and the size of the infarction, assessed by magnetic resonance imaging - the rationale is that delayed stent may allow time for the action of antithrombotic drugs, reduced thrombus burden and consequently less NR and smaller infarcted area. In fact, in this study, there was a significant reduction in NR (from 14% to 2% in the stent-delayed group) and an improvement in the myocardial salvage rate in 6 months.^12^ Later, in 2017, a meta-analysis brought together 9 studies and the reduction in NR was not observed, however, an improvement in long-term ventricular function in the delayed stent group was pointed out.^13^

Other strategies such as the use of intracoronary drugs (adenosine, calcium channel blockers and nitroprusside) and glycoprotein IIb/IIIa inhibitors have shown some benefit in the prevention and treatment of NR and need further studies.^14^

The NR phenomenon is the greatest challenge of primary reperfusion and despite efforts; knowledge has evolved little in the treatment or prevention of this condition. Angiographic scores, which make the assessment of cineangiocoronariography more objective, contribute to the discrimination of patients with a worse prognosis, particularly when the association of classic scores is used (SYNTAX, Gensini, Thrombotic load), as demonstrated in this article. Recognizing these patients can lead to optimization of treatment and improvement of outcomes.
